# Acute cardiovascular and muscular response to rowing ergometer exercise in artificial gravity – a pilot trial

**DOI:** 10.1038/s41526-024-00402-7

**Published:** 2024-05-23

**Authors:** Timo Frett, Leo Lecheler, Michael Arz, Willi Pustowalow, Guido Petrat, Florian Mommsen, Jan Breuer, Marie-Therese Schmitz, David Andrew Green, Jens Jordan

**Affiliations:** 1 Institute of Aerospace Medicine, German Aerospace Center, Cologne, Germany; 2 Institute of Medical Biometry, Informatics and Epidemiology, Medical Faculty, University of Bonn, Bonn, Germany; 3 European Space Agency, Cologne, Germany; 4 King’s College London, London, UK; 5 Space Medicine Team, European Astronaut Centre, European Space Agency, Cologne, Germany; 6 KBRwyle GmbH, Cologne, Germany; 7 Chair of Aerospace Medicine, University of Cologne, Cologne, Germany

**Keywords:** Medical research, Risk factors

## Abstract

Prolonged immobilization and spaceflight cause cardiovascular and musculoskeletal deconditioning. Combining artificial gravity through short-arm centrifugation with rowing exercise may serve as a countermeasure. We aimed to compare the tolerability, muscle force production, cardiovascular response, and power output of rowing on a short-arm centrifuge and under terrestrial gravity. Twelve rowing athletes (4 women, aged 27.2 ± 7.4 years, height 179 ± 0.1 cm, mass 73.7 ± 9.4 kg) participated in two rowing sessions, spaced at least six weeks apart. One session used a short-arm centrifuge with +0.5 Gz, while the other inclined the rowing ergometer by 26.6° to mimic centrifugal loading. Participants started self-paced rowing at 30 W, increasing by 15 W every three minutes until exhaustion. We measured rowing performance, heart rate, blood pressure, ground reaction forces, leg muscle activation, and blood lactate concentration. Rowing on the centrifuge was well-tolerated without adverse events. No significant differences in heart rate, blood pressure, or blood lactate concentration were observed between conditions. Inclined rowing under artificial gravity resulted in lower power output (−33%, *p* < 0.001) compared to natural gravity, but produced higher mean and peak ground reaction forces (*p* < 0.0001) and increased leg muscle activation. Muscle activation and ground reaction forces varied with rotational direction. Rowing in artificial gravity shows promise as a strategy against cardiovascular and muscular deconditioning during long-term spaceflight, but further investigation is required to understand its long-term effects.

## Introduction

Spaceflight poses several physiological challenges to the human body, including alterations in cardiovascular function^1,2^, muscle and bone loss^3^, and sensory-motor changes^4,5^. Despite implementing daily resistance and aerobic exercise countermeasures on the International Space Station^6^, microgravity-induced physiological deconditioning is evident^7^, though its extent varies^8^. While problematic upon re-entry to Earth, this deconditioning could prove critical when landing on lunar or Martian surfaces^9^. Consequently, more efficient multi-organ countermeasures are necessary for future exploration missions^10^. The application of artificial gravity through short-arm human centrifugation, generating centripetal acceleration to simulate gravitational effects, has been suggested to counteract multi-system deconditioning^11^. Although passive Artificial Gravity with 1 g at the Center of Mass has been demonstrated tolerability^12^, daily use yields relatively low physiological load^13^, possibly explaining its low effectiveness against bed rest-induced multi-system deconditioning^14,15^. Exercise during short arm centrifugation might offer better results. However, head movement during short arm centrifugation is associated with disorientation and motion sickness^16^ and orthostatic intolerance can occur^17^. Nevertheless, with moderate gravity load (e.g., 1 g at center of mass) and congruent head and body motion, moderate movement during concurrent plyometric exercises^18^ and trunk exercise^19,20^ is well-tolerated. During movement along the centrifuge radius, lateral differences depending on rotational direction were found resulting in an asymmetric loading of left and right legs^21^. Hemodynamic responses to these exercises seem largely similar to those on Earth, albeit at relatively low intensities. Additionally, whilst plyometric exercises such as jumping can be performed during short arm centrifugation, familiarization is required to replicate generation of equivalent reaction forces to ground conditions^22,23^ High-intensity whole-body exercises are preferable for spaceflight to counter muscle and bone loss and cardiovascular deconditioning^24,25^. One such exercise protocol is rowing ergometry, which is an effective exercise modality for promoting cardiorespiratory fitness and whole-body strength on Earth with moderate impact forces^26,27^. Currently, the only device on the International Space Station allowing rowing exercises is the miniature exercise device-2 (MED-2)^28,29^, which simulates rowing through a motor-controlled pulley. However, crew members are unable to fully replicate terrestrial rowing strokes as the device prevents them from extending beyond their feet^30^. Rowing ergometry may be well-suited for use during centrifugation due to its low-impact nature on joints and ability to generate a large muscle mass and cardiovascular load. A challenge, however, are the Coriolis forces and g-gradient´s effect on the cardiovascular system^31^ and, potentially, on rowing biomechanics during eccentric and concentric movements along the centrifuge radius. As rotational direction can lead to one-sided strain^23^ we compared both centrifuge directions (clockwise and counter-clockwise) in randomized order. Therefore, this pilot trial assessed exercise load in terms of intensity (cardiopulmonary response, leg muscle activation, ground reaction forces, and rowing performance) and tolerability (motion sickness scores and perceived exertion) when performed on a centrifuge in different rotational directions in comparison to rowing in 1 g.

## Results

### Gravitational loading and compliance

To generate the individualized +0.5 Gz at midpoint position during a rowing stroke, the average centrifuge spin rate was 15.4 ± 0.4 rpm at a distance of 245 ± 3.7 cm from the rotational axis. All participants completed the exercise sessions without presyncope, emesis, pain, or other clinically relevant issues. Based on our prospectively defined criteria, rowing exercise in artificial gravity was feasible, well tolerated, and provided a partial greater physiological stimulus than rowing in terrestrial gravity.

### Cardiovascular response

**Fig. 1:**
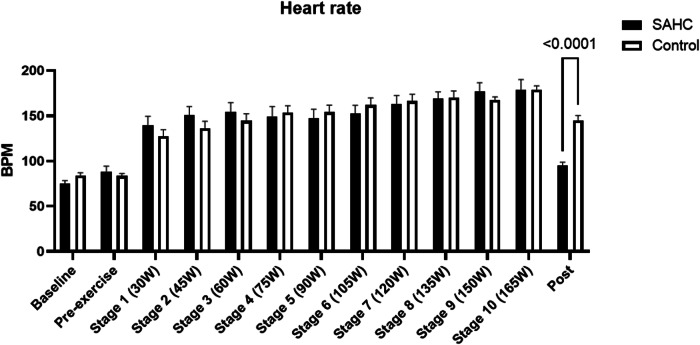
Heart rate at baseline (without centrifugation), prior exercise (Pre-exercise), during gradually increased rowing and after end of rowing (POST) for both groups. Mean ± SD.

Heart rate (Fig. 1) significantly increased over time during self-paced rowing (F (3.010, 40.14) = 123.0, *p* < 0.0001) but without differences between conditions (F (1, 21) = 0.4599, *p* = 0.50). Maximal achieved heart rate values were 181.2 ± 9.8 BPM during SAHC and 185.5 ± 9.0 BPM during CONTROL.

**Fig. 2:**
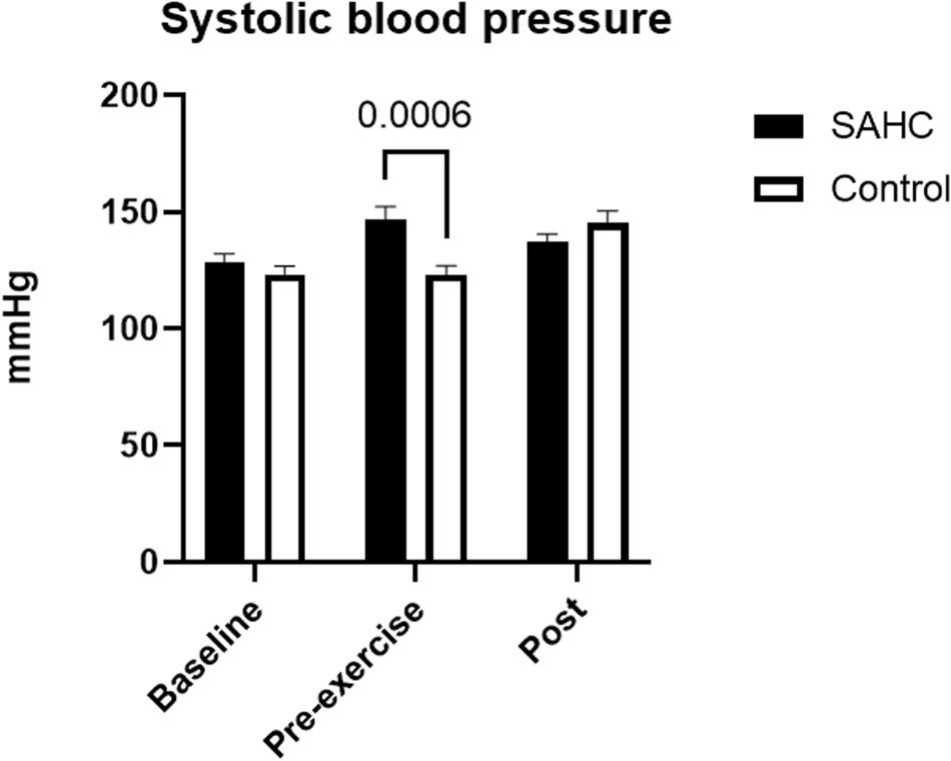
Systolic blood pressure at baseline, prior exercise and after end of rowing. Mean ± SD.

**Fig. 3:**
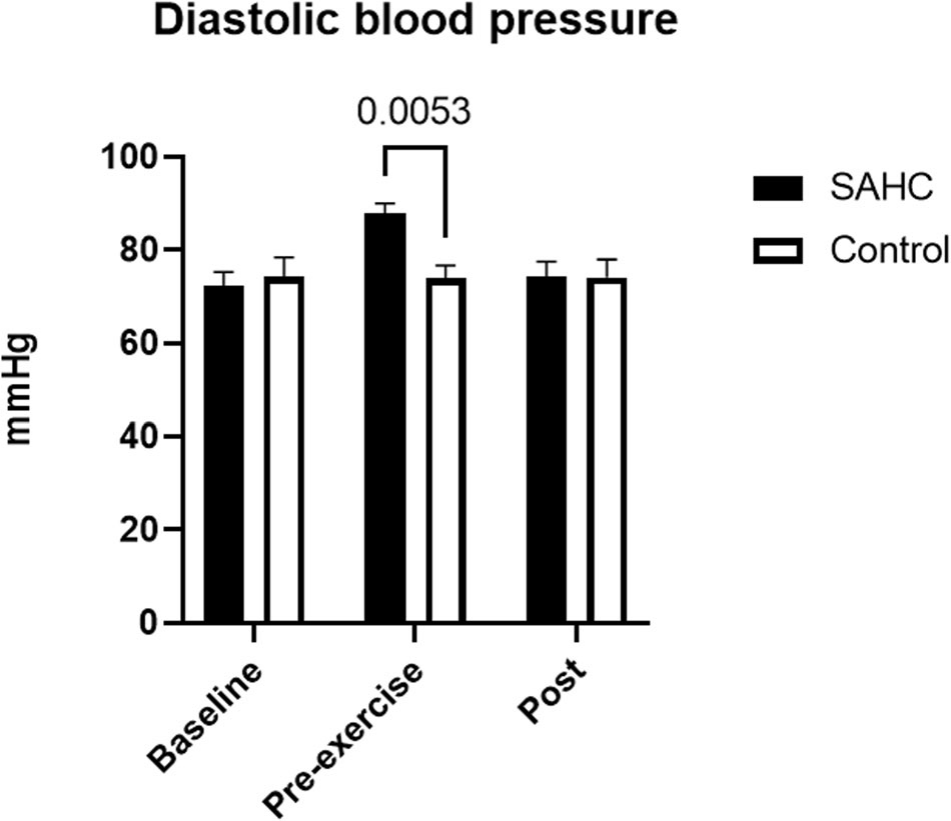
Diastolic blood pressure at baseline, prior exercise and after end of rowing. Mean ± SD.

Blood pressure values (Fig. 2, Fig. 3) showed a significant interaction effect (time x condition, systolic: *p* = 0.0015, diastolic: *p* = 0.0058) and were higher prior exercises (Pre-exercise) during SAHC compared to CONTROL (systolic: *p* = 0.0006, diastolic: *p* = 0.005) but not after rowing (systolic: *p* = 0.65, diastolic: *p* = 0.99).

### Blood lactate

**Fig. 4:**
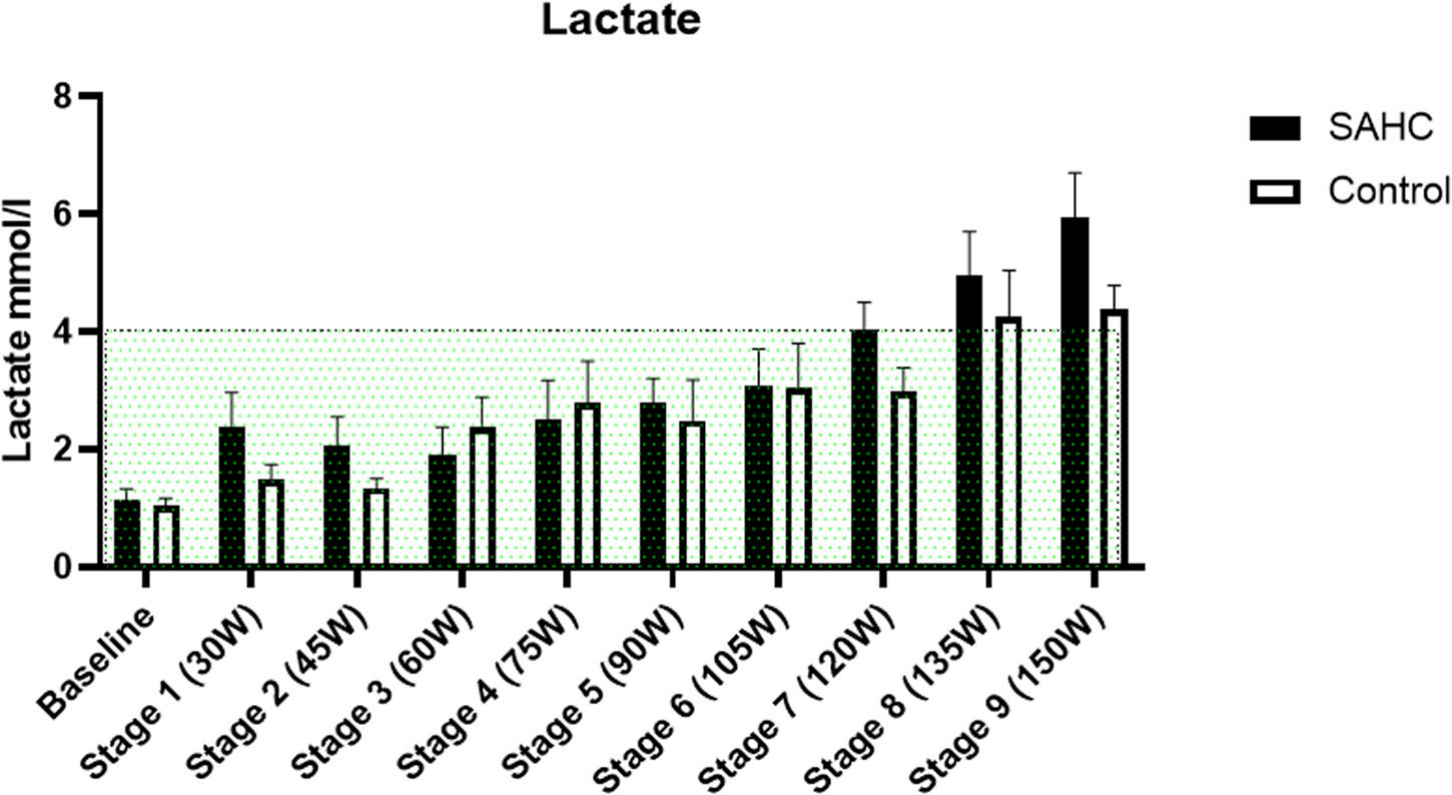
Blood lactate during baseline and after each rowing stage of self-paced rowing during short arm centrifugation (SAHC) and in Earth terrestrial control (Control). Anaerobic threshold (>4 mmol/l) marked in green. Mean ± SD.

Blood lactate concentrations (Fig. 4). obtained by blood samples during baseline and after each stage of self-paced rowing increased over time (F (9, 103) = 6.002, *p* < 0.0001) but did not differ between conditions (F (1, 103) = 2.635, *p* = 0.11). Participants reached the anaerobic threshold defined at >4 mmol/l lactate in both conditions.

### Cardioventilatory responses

All cardioventilatory parameter obtained during the incremental self-paced rowing test until exhaustion are shown (Table 1). We did not observe a significant effect of condition for VT1 and VT2. All participants reached calculated VO_2_max in each condition. The only significant effect of condition found was for VO2max (F (1, 252) = 15.30, *p* = 0.0001) with higher ventilation (V’E) in control group (SAHC: 108.03 ± 20.7, CONTROL: 128.97 ± 22.6; *p* < 0.0001). No other effects of condition were noted (*p* > 0.05).

**Table 1:** Cardioventilatory parameter for VT1, VT2, and VO2max. Data provided as mean ± standard deviation (SD) and percentage (%)

Variable	Condition	VT1	*p*	VT2	*p*	VO_2max_	*p*
AF (/min)	AG CTRL	31.00 ± 9.64 33.09 ± 6.83	>0.999	37.50 ± 5.17 39.80 ± 4.61	0.9994	47.83 ± 6.28 52.45 ± 9.14	0.8818
VO2 (L/min)	AG CTRL	2.03 ± 0.40 2.16 ± 0.74	>0.999	2.45 ± 0.53 2.78 ± 0.66	>0.9999	2.92 ± 0.70 3.42 ± 0.86	>0.9999
V’O2/kg (ml/min/kg)	AG CTRL	27.92 ± 5.93 29.00 ± 8.63	>0.999	33.30 ± 6.41 37.20 ± 7.24	0.9459	39.50 ± 7.86 45.55 ± 9.75	0.5711
V’CO2 (L/min)	AG CTRL	1.79 ± 0.55 1.90 ± 0.79	>0.999	2.46 ± 0.48 2.73 ± 0.56	>0.9999	3.01 ± 0.64 3.54 ± 0.76	>0.9999
RER	AG CTRL	0.91 ± 0.07 0.85 ± 0.15	>0.999	1.01 ± 0.07 0.99 ± 0.06	>0.9999	1.04 ± 0.05 1.04 ± 0.05	>0.9999
V’E (L/min)	AG CTRL	59.02 ± 20.45 62.99 ± 20.12	>0.999	82.33 ± 18.15 88.78 ± 15.89	0.3961	108.03 ± 20.71 128.97 ± 22.63	<0.0001
V’E/V’O_2_	AG CTRL	27.40 ± 3.91 26.14 ± 1.95	>0.999	31.33 ± 4.11 30.09 ± 2.48	>0.9999	34.93 ± 4.41 35.85 ± 3.44	>0.9999
V’E/V’CO_2_	AG CTRL	29.94 ± 2.59 29.44 ± 1.75	>0.999	30.90 ± 2.73 30.32 ± 1.57	>0.9999	33.58 ± 2.99 34.35 ± 2.05	>0.9999
PetO_2_ (mmHg)	AG CTRL	109.75 ± 5.26 109.00 ± 2.61	>0.999	114.2 ± 3.97 112.60 ± 3.31	>0.9999	117.75 ± 4.22 118.55 ± 2.98	>0.9999
PetCO_2_ (mmHg)	AG CTRL	36.42 ± 2.27 39.09 ± 2.95	>0.999	35.80 ± 2.78 38.80 ± 2.49	0.9933	33.25 ± 2.80 34.82 ± 2.60	>0.9999
HF (/min)	AG CTRL	143.42 ± 24.89 146.18 ± 16.65	>0.999	166.60 ± 16.59 171.70 ± 9.58	0.7389	177.50 ± 11.19 185.82 ± 7.90	0.1387
%VO_2peak_ (%)	AG CTRL	68.50 ± 17.92 59.55 ± 16.00	0.4041	84.00 ± 11.87 83.30 ± 6.00	>0.9999	99.83 ± 1.64 99.55 ± 2.54	>0.9999

*HR* heart rate, *RER* respiratory exchange ratio, *VCO*_*2*_ carbon dioxide, *V’E* minute ventilation, *V’E/V’CO*_*2*_ ventilatory equivalent for carbon dioxide, *VE/VO*_*2*_ ventilatory equivalent for oxygen, *VO*_*2*_ oxygen uptake, *%VO*_*2*_*peak* relative peak oxygen uptake, *VT1* first ventilatory threshold, *VT2* second ventilatory threshold, *VO*_*2*_peak peak oxygen uptake.

### Rowing performance

**Fig. 5:**
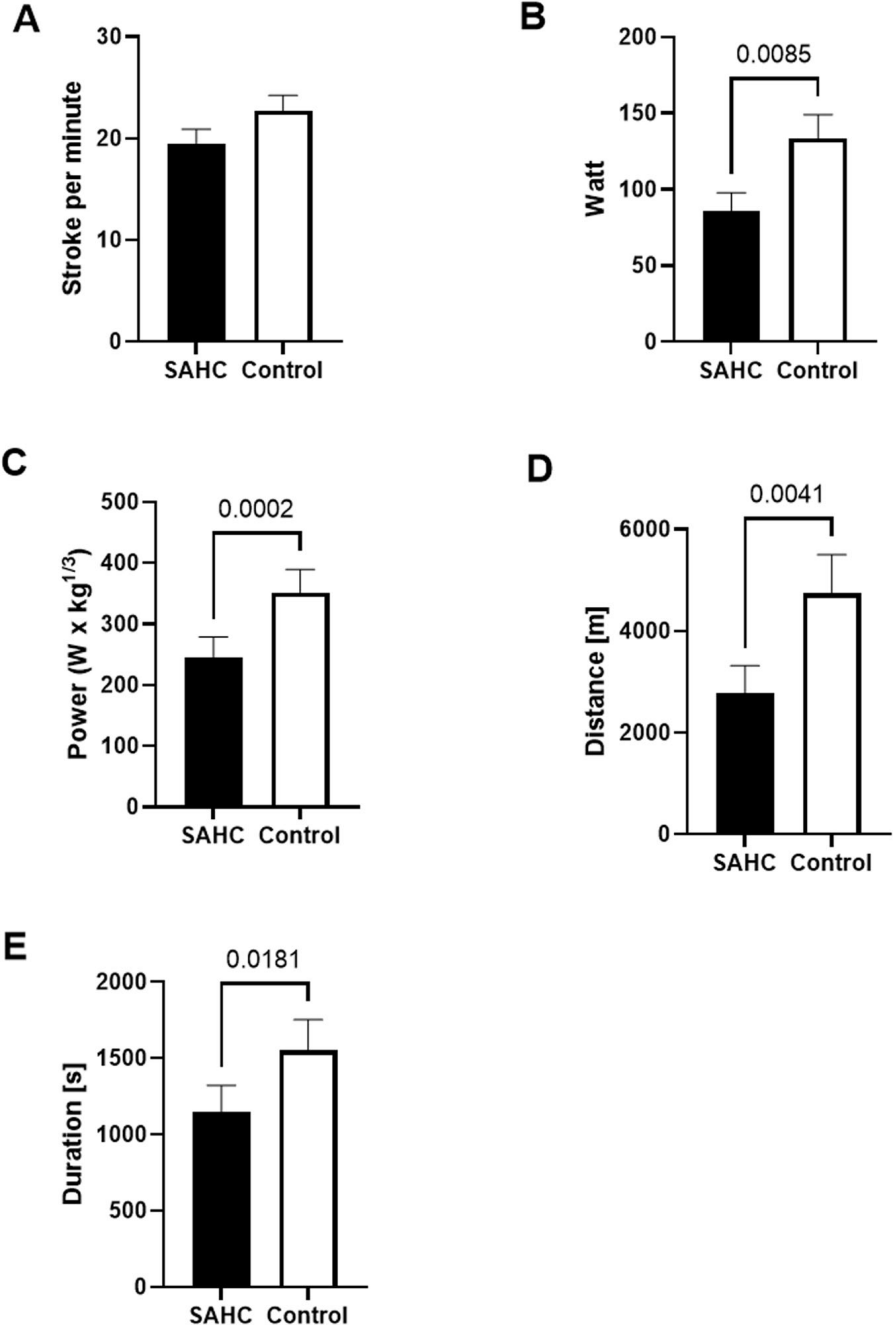
Comparison of rowing performance during short arm centrifugation (SAHC) and in Earth terrestrial control (Control). Average stroke rate per minute (**A**), average wattage (**B**), calculated power (W· kg^1/3^ Watt per corrected body mass) (**C**), rowing distance (**D**), rowing duration (**E**). Mean ± SD.

Rowing performance (Fig. 5, Table 2) during centrifugation was significantly lower for average wattage (*p* < 0.0001), power [W x k1/3] (*p* = 0.0012), maximal distance (*p* = 0.003) and maximal duration (*p* = 0.024) compared to CONTROL whilst average stroke rate was maintained (*p* = 0.16). Maximal achieved workload was 85.7 ± 38.8 W during SAHC and 128.6 ± 51.7 W during control (−33%, *p* < 0.001).

**Table 2:** Comparison of perfomance between rowing on the short arm human centrifuge and Earth terrestrial control

	SAHC	CONTROL	Paired *t* test
Stroke rate (spm)	20.0 ± 1.5	23.1 ± 2.0	t (6) = 1.606, *p* = 0.16
Watt (W)	61.4 ± 7.9	88.3 ± 7.8	t (6) = 8.616, ***p < 0.0001
Power (W x kg^1/3^)	182.0 ± 46.1	363.6 ± 38.8	t (9) = 4.678, **p = 0.001
Total distance (m)	3006 ± 652.8	5135 ± 839.0	t (7) = 4.402, **p = 0.003
Total duration (s)	1202 ± 193.9	1761 ± 232.0	t (6) = 2.986, *p = 0.024

*SAHC* Short arm human centrifuge, *Control* Earth terrestrial control, *spm* strokes per minute, *W· kg1/3* Watt per corrected body mass, *s* seconds, *m* meter.

*p < 0.05; ***p < 0.001. Mean ± SD.

### Force plate data

Ground reaction forces during rowing exhibited the highest mean values for SAHC-CW, followed by SAHC-CCW and CONTROL (F (2, 244) = 258.935, *p* < 0.001). Similarly, peak ground reaction forces were notably higher for SAHC-CW (*p* < 0.001) and SAHC-CCW (*p* < 0.001) compared to rowing without centrifugation, displaying an increase over time (*p* < 0.001). This trend was consistent during both the concentric and eccentric phases of rowing, with mean ground reaction forces being higher for SAHC-CW (*p* < 0.001) and SAHC-CCW (*p* < 0.001) in comparison to the control condition.

**Fig. 6:**
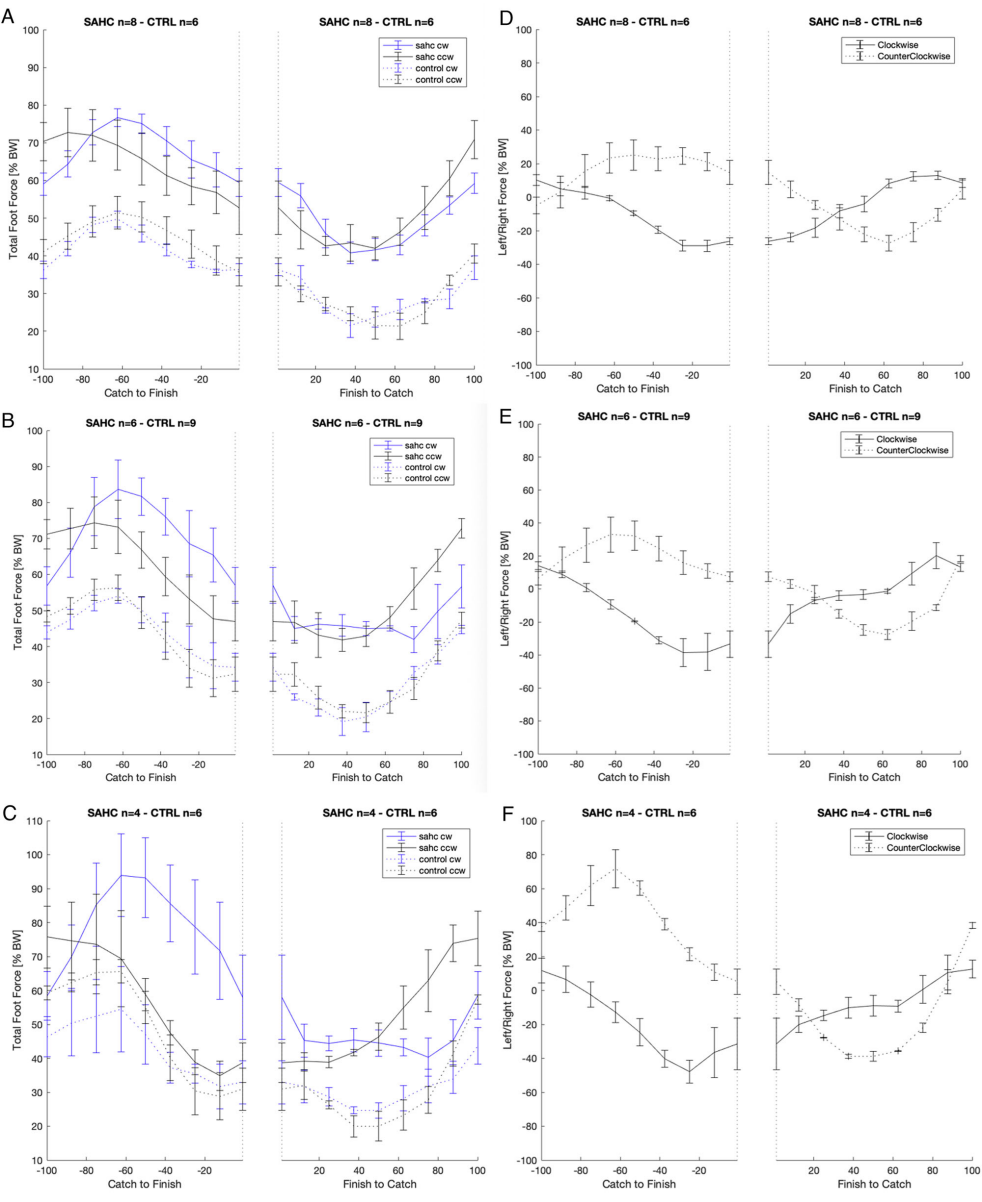
Ground reaction forces depending on rowing motion and rotational direction on the centrifuge. Left: Mean total foot force (as percentage of body weight) for matched pairs of participants as function of rowing motion for leg extension (catch to finish) and leg flexion (finish to catch) for rowing at low intensity (**A**, stage 1: 30 W), moderate intensity (**B**, stage 4: 75 W) and high intensity (**C**, stage 8: 135 W). Mean ± SEM. Right: Ratio left to right foot force (as percentage of body weight) depending on rotational direction during centrifugation for rowing at low intensity (**D**, stage 1: 30 W), moderate intensity (**E**, stage 4: 75 W) and high intensity (**F**, stage 8: 135 W). Mean ± SEM.

Rotational direction during centrifugation played a role in lateral loading, specifically rotation x side interaction. An interaction effect (F (2, 239) = 85.321, *p* < 0.0001) between rotational direction and lateral loading was observed. In concentric phase, mean foot forces were higher on the right foot during SAHC-CW, while higher foot forces were recorded on the left foot during SAHC-CCW. This pattern was consistent during both the concentric (F (2, 241) = 144.374, *p* < 0.0001) and eccentric (F (2, 241) = 46.510, *p* < 0.0001) phases and followed an approximate sinusoid during the rowing stroke (Fig. 6).

### Muscle activitiy

**Fig. 7:**
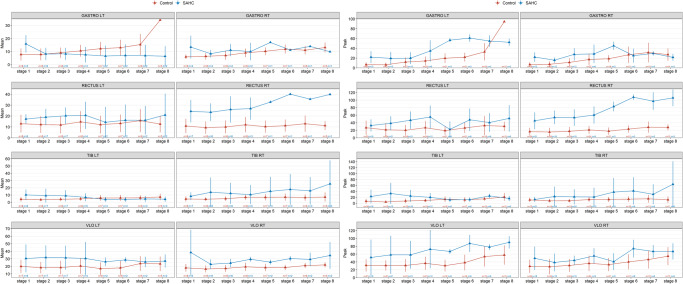
Comparison of leg muscle activity during centrifugation and control condition. Left: Bilateral values (left and right leg) for mean muscle activation as percentage of maximum voluntary contraction for rowing during centrifugation (SAHC) and without rotation (control). Mean ± SD. Right: Bilateral values (left and right feet) for peak ground reaction forces for rowing during centrifugation in clockwise rotation (SAHC-CW), counter clockwise rotation (SAHC-CCW) and without rotation (control). Mean ± SD.

We observed a significant effect for the condition (F (1, 672) = 82.690, *p* < 0.0001) and a significant 2-way interaction (muscle x condition) effect (F (7, 672) = 8.544, *p* < 0.0001). There was a notably higher mean activation (%MVC) during centrifugation (Fig. 7) for specific muscles: rectus femoris (left: +37.1%, *p* = 0.002, right: +183.7%, *p* < 0.001), tibialis (left: +23,4%, *p* = 0.04, right: +147%, *p* < 0.001), and vastus lateralis (left: +45,2%, *p* < 0.001, right: +55%, *p* < 0.001), although not for gastrocnemius (left: −39%, *p* = 0.07, right: −27,1%, *p* = 0.06). Additionally, as rowing intensity increased, we noted a corresponding rise in average (F (1, 672) = 11.609, *p* = 0.007) and peak muscle activation (F (1, 1872) = 170.053, *p* < 0.0001). Peak activation (Fig. 7) was significantly greater during centrifugation for rectus femoris (left: +65%, *p* = 0.003, right: +263.8%, *p* < 0.001) and left vastus lateralis (+218%, *p* < 0.001), but not for right vastus lateralis (*p* = 0.13), tibialis (left: +84%, *p* = 0.34, right: +167.1%, *p* = 0.13), and gastrocnemius (left: +101.2%, *p* = 0.72, right: +47%, *p* = 0.91).

We observed significantly greater activation of leg muscles during rowing along the centrifuge radius for both concentric movements (F (1, 1872) = 362.289, *p* < 0.0001) with increased activation in the left rectus femoris (*p* = 0.01), left tibialis (*p* = 0.002), and left vastus lateralis (*p* = 0.01). This pattern was also evident for eccentric movements (F (1, 1872) = 542.137, *p* < 0.0001) in rectus femoris (left: *p* < 0.001, right: *p* < 0.001) and vastus lateralis (left: *p* = 0.005, right: *p* < 0.001).

### Questionnaires

Motion sickness scores remained low (<5) in all conditions but displayed a significantly greater increase over time during SAHC (t (11) = 3.272, *p* = 0.007) compared to the CONTROL condition (t (9) = 1.984, *p* = 0.08). However, when directly comparing the total motion sickness scores after rowing (PRE vs POST), no significant differences emerged between the two conditions (t (9) = 0.9190, *p* = 0.38). Similarly, an analogous increase was observed for MSAQ ratings during SAHC (t (11) = 2.455, *p* = 0.03), but not during the CONTROL condition (t (9) = 1.225, *p* = 0.25), with no notable condition effect (t (9) = 0.05491, *p* = 0.96).

The rate of perceived exertion increased in both conditions over time (SAHC: t (11) = 12.62, *p* < 0.0001, CONTROL: t (9) = 8.476, *p* < 0.0001) without a significant difference between conditions at the end of rowing (SAHC: 15.8 ± 0.7, CONTROL: 15.6 ± 1.0; t (9) = 0.3094, *p* = 0.76). Notably, no significant differences were observed for PANAS or ESS assessments.

## Discussion

The important finding of this this pilot study is that rowing in artificial gravity is well-tolerated and feasible. Furthermore, rowing in artificial gravity results in increased muscular effort due to higher muscle activation and ground reaction forces compared to rowing under normal gravity. However, the power output was reduced by a third during centrifugation. Cardiovascular responses to rowing were similar between conditions, but rowing on the centrifuge exhibited greater musculoskeletal effort and potential for improved training stimulus. The strength of our study is that we conducted a comparative analysis during self-paced rowing on a short-arm centrifuge and in an inclined position under terrestrial gravity, which allows to dissect out influences of artificial gravity on adverse effects and cardiovascular as well as muscular loads during rowing exercise.

Motion sickness, a concern in artificial gravity, was minimal in participants performing rowing strokes in this setup. In our pilot trial, participants executed complete rowing strokes and had the freedom to move their head, torso, arms, and legs. Safety belts secured only the hips and feet to avoid injuries during centrifugation. Notably, head movements while rotating in a steep gravitational gradient on a centrifuge are known to generate cross-coupling stimuli that may induce vestibular sensations such as tumbling and nausea^11,16^. The widely accepted sensory mismatch theory^32–34^ posits that sensory conflicts are perceived as nauseogenic when the actual sensory pattern significantly deviates from the brain’s expectations based on prior experiences^16^. However, human beings can adapt to cross-coupled forces while lying passively after frequent exposure to short arm centrifugation^12,35,36^. This reduced susceptibility to motion sickness likely results from the shortened time constants in velocity storage mechanisms during neurovestibular processing^37,38^. Additionally, our previous findings suggested, that exercises involving repetitive head movements, such as jumping on a centrifuge, are well tolerated^18,39^. Our pilot trial also demonstrated that rowing during centrifugation was well tolerated, with no sessions being interrupted due to motion sickness. This may be attributed to the fact that exercising on a centrifuge reduces the typical sensory mismatch caused by unfamiliar vestibular inputs, thanks to additional afferent proprioceptive information from muscle spindles (type Ia and II neurons), Golgi tendons (type Ib neurons), or joint receptors (Ruffini endings and Pacinian corpuscles)^40^. These proprioceptive patterns generally align with past sporting experiences, aiding the brain in predicting accelerations based on participants’ movements. Similar mechanisms are known from road vehicles, where the driver is able to predict low-frequency horizontal accelerations and can carry out compensatory actions to reduce motion sickness whereas passengers are more likely prone to motion sickness symptoms^16^.

Though well tolerated, rowing during centrifugation appeared to be more demanding in terms of rowing performance, with no significant differences in cardiovascular and ventilatory parameters between conditions. Transitioning from upright seated in normal gravity to sitting while rotating with +0.5 Gz induced a greater increase in blood pressure compared to sitting in an inclined position, indicating a stronger influence of caudal fluid shift. During rowing, heart rate increased comparably with rising wattage in both conditions. After the cessation of rowing exercise, heart rate remained elevated in the control condition, primarily due to lower caudal hydrostatic pressure following centrifugation stoppage and reconditioning in an upright seated position. Though ventilatory parameters and thresholds (VT1, VT2) remained largely unaffected by centrifugation compared to normal gravity, greater muscle activation in rectus femoris, tibialis anterior, and vastus lateralis during rowing indicated an increased musculoskeletal effort likely aimed at counteracting lateral loading and gravity gradient. Furthermore, Coriolis accelerations are known to cause asymmetric volume changes between legs^41^. This observation is supported by force plate data demonstrating higher peak loads depending on rotational direction. Clockwise centrifugation and rowing without rotation resulted in higher peak loads on the right foot, while counter clockwise centrifugation led to higher peak loads on the left foot. As all participants were right-foot dominant, the rotational direction seemed to directly influence lateral loading in a short-radius setup. During centrifugation, the left to right foot difference followed an approximate sinusoid. This influence persisted consistently throughout both the concentric and eccentric phases of rowing cycles and was also observed as a tendency during plyometric^23^ and squat exercises on a centrifuge^21^. Furthermore, higher mean and peak values of ground reaction forces during centrifugation indicated the impact of the force gradient while moving back and forth along the centrifuge radius while rowing. As we had individually determined the g-reference in the middle position of a rowing stroke, participants crossed this point during exercise. As a result, the highest g-load was experienced at the catch position, where the rower leans forward the most, requiring increased force development. Taken together, increased lateral loading and the effects of the force gradient likely result in higher muscular effort, making rowing on a centrifuge more demanding in terms of a shorter time until exhaustion. Additionally, though blood lactate concentrations were not significantly higher during rowing on the centrifuge, they exhibited a tendency to exceed the rate at which lactate breaks down post-exercise which usually indicates greater muscular loading. Our study has some limitations. As we tested, to our knowledge, for the first-time tolerability and efficacy of rowing during centrifugation, we included only twelve participants. Nevertheless, this size was adequate for estimating the tolerability and overall feasibility of rowing on a centrifuge. To comprehensively evaluate the impacts of rowing on the cardiovascular and musculoskeletal systems within a short-radius centrifuge setup, larger groups, and additional repetitions would be necessary. Another aspect is the mass of the rowing seat. To ensure safety of all participants, a rowing seat with a solid back plate was used with a mass of 18 kg. Although this seat was identical in both conditions it certainly lowered participants rowing performance.

In conclusion, our pilot study demonstrates that rowing within a moderate g-level of +0.5 Gz during centrifugation is feasible and well tolerated. Furthermore, rowing ergometry in artificial gravity appears to provide a greater physiological stimulus compared to rowing ergometry in normal gravity due to lateral effects and a force gradient. These results may also have practical implications for countermeasure development in space and for athletes on Earth. Further studies with larger sample sizes and more diverse populations are necessary to confirm these findings and evaluate the potential long-term benefits of rowing ergometry in artificial gravity.

## Methods

### Participants

We studied twelve rowing athletes (4 women, 8 men), with a mean age of 27.2 ± 7.4 years, a height of 179 ± 0.1 cm, and a mass of 73.7 ± 9.4 kg. The athletes attended the laboratories at the :envihab (German Aerospace Center (DLR), Cologne, Germany) on two occasions, separated by at least six weeks to minimize the risk for a carry-over effect. Before participating in the study, all participants gave written informed consent, completed a brief medical questionnaire, and underwent a standardized centrifuge medical screening, which included clinical-chemical analyses of blood and urine, stress electrocardiogram, and orthostatic testing. We excluded participants with acute pain or any significant current or past musculoskeletal, cardiovascular, or neurological disorder or injury that could affect their ability to perform exercise from the study. No anti-emetic medication was allowed and participants were provided with light food (cereal bars) and non-sparkling water prior testing *ad libitum* to ensure adequate hydration and glycemia. All participants were right leg dominant. The North Rhine Medical Association Ethics Committee approved the study. The study was prospectively registered in a public database (German Clinical Trials Register; DRKS00021750).

### Study design and protocol

**Fig. 8:**
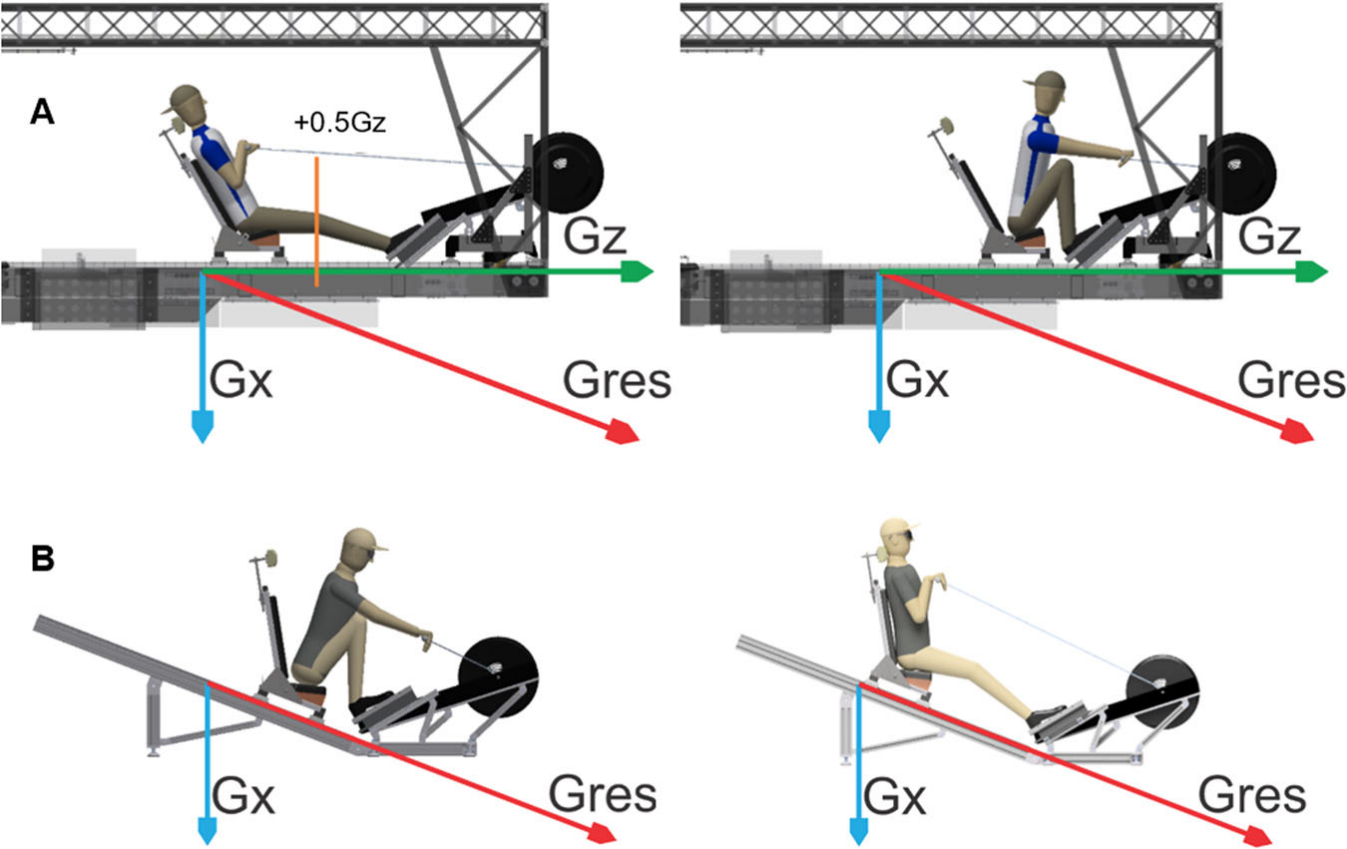
Schematic overview about the exercise setups for each condition. Drawings of the rowing installation on the centrifuge (**A**) and in inclined position under terrestrial gravity (**B**) with acceleration vectors for Earth´s gravity (Gx), centrifugal acceleration (Gz) and resulting vector (Gres). Participants were able to freely move back and forth for a rowing stroke. On the centrifuge, we individually determined the +0.5 Gz by measuring the midpoint for a complete rowing stroke (**A**: orange line).

Participants performed rowing exercises on an indoor rowing ergometer (Concept2 Inc., Vermont, USA) that was placed either on a centrifuge with +0.5 Gz in clockwise or counterclockwise direction (SAHC) or in a 26.6° inclined position under terrestrial gravity (CONTROL) in a randomized crossover fashion on separate days. The cyclical motion of the rowing stroke is divided in four phases: catch, drive, finish, and recovery^42^. Since the centripetal acceleration changes linearly with the distance from the center of rotation, we individually determined the necessary angular velocity on the centrifuge before the experiment. This was accomplished by having each participant move back and forth along the centrifuge radius to measure the midpoint for a complete rowing stroke. We then calculated inclination of the rower under terrestrial gravity to match the resulting acceleration vector during centrifugation with +0.5 Gz at the described reference point. Safety regulations required a rowing seat with solid back plate weighing 18 kg that was inclined rearwards by 30° to ensure correct upper body movement during rowing. Participants were secured by safety belts around the hip and feet but could freely move their upper body, legs, and arms for rowing Fig. 8.

Before the first session, participants were familiarized with the equipment, testing procedures, and exercises. Each session included resting measurements in the supine position (BASELINE), immediately prior to exercises (PRE-EXERCISE), and after exercise (POST). We asked participants to complete as many intensity levels as possible during self-paced rowing, starting at 30 W, with gradual increases of 15 W every three minutes until exhaustion. We recorded rowing performance, heart rate, brachial blood pressure, ground reaction forces, spirometry, leg muscle activation, Motion Sickness rating, rate of perceived exhaustion and obtained blood samples from an antecubital venous catheter to measure blood lactate concentration.

### Heart rate and blood pressure

We continuously recorded heart rate via a five-lead electrocardiogram and discontinuously measured oscillometric brachial blood pressure (Philips IntelliVue® MP2, Eindhoven, The Netherlands). On the centrifuge, we recorded blood pressure at BASELINE, after achieving +0.5 Gz (PRE-EXERCISE), and 15 min after completion of exercises and stop of centrifugation (POST). During rowing in terrestrial gravity, we recorded blood pressure at BASELINE, during inclined sitting prior rowing (PRE-EXERCISE), and 15 min after completion of exercises (POST).

### Blood lactate concentration

Blood samples were collected using an antecubital venous catheter and a mobile lactate measurement device (ACCUTREND Plus Roche, Switzerland) at the end of each intensity level to measure blood lactate concentrations. During centrifugation, an operator placed on a chair on the rotational axis obtained blood samples for lactate measurements from the participant while spinning to enable continuous rowing.

### Spiroergometry

Prior and during rowing at increasing loads, we determined maximal aerobic power using a breath-by-breath spiroergometric system (Cortex Biophysik GmbH, Germany). Before each trial, the system was calibrated. We assessed ventilatory thresholds (VT1, VT2), maximal oxygen consumption (VO2max), respiratory exchange ratio, and ventilation. VT1, the first ventilatory threshold, was determined using Wasserman’s V-slope method, identifying the breakpoint in the VCO2–VO2 relationship (VCO2 representing carbon dioxide production and VO2 representing oxygen consumption). VT2 was determined in two ways: (1) as the second rise in ventilation; (2) as the intensity accompanying a second rise in the VE/VO2 relationship with a concurrent rise in the VE/VCO2 relationship. VO2max was considered achieved if two of the following three criteria were met: heart rate ≥95% of the theoretical maximum (calculated as 220 minus age), RER (respiratory exchange ratio) ≥1.10, and a plateau in VO2 despite increasing exercise intensity.

### Rowing performance

We collected rowing data using the installed PM5 Performance Monitor (Concept2 Inc., Vermont, USA). The monitor delivered individual workout data including stroke rate, average watts, power (corrected with body mass), total rowing distance, and duration with an error ranging from 0.2 to 1.9% once the flywheel has been accelerated^43^.

### Ground reaction force

We measured ground reaction forces during rowing at 1000 Hz using force plates (AMTI, USA) below the left and right foot. We distinguished force plate data between clockwise and counterclockwise centrifugation. Local peaks were identified and for every stage 5 consecutive strokes were averaged. Rowing produces a cyclical motion comprising four phases designated as catch, drive, finish, and recovery^42^. We separated force plate data into concentric motion (Finish to Catch) and eccentric motion (Catch to Finish).

### Trunk muscle surface electromyography

We applied surface electromyography (EMG) to assess trunk muscle activity during rowing. We placed bipolar telemetric surface electrodes (Noraxon Ultium, USA) bilaterally on the rectus femoris, vastus lateralis, gastrocnemius, and tibialis anterior muscles after shaving, exfoliating, and cleaning the skin with alcohol. We sampled EMG signals at 2000 Hz and bandpass filtered between 10 and 500 Hz. Furthermore, we marked start and end of individual intensity onsets and offsets of EMG activity and applied root mean square (RMS) filter (100 ms window). Prior to each experiment, participants performed three maximum voluntary contractions (MVC) of each muscle group^44^ with at least a 1-min rest interval between maximal efforts. We used the recorded MVCs to normalize subsequent EMG signals (%MVC), which were averaged (left and right) per muscle.

The recording period between reaching the required centrifugal speed and the first rowing stroke was averaged per participant and taken as the baseline. Two standard deviations above the baseline count as muscle activation. During rowing, we extracted five consecutive rowing strokes (catch to catch) from each activity 30 s after the onset of a new intensity level. Furthermore, we converted each stroke to 100% in the time domain to allow comparison of muscle activation.

### Motion sickness questionnaires and subjective exertion rating

To assess susceptibility to motion sickness and exertion, participants completed a short-form motion sickness susceptibility questionnaire (MS: 0 = “I am feeling fine” to 20 = “I am about to vomit”)^36^ and perceived exertion questionnaire (RPE: 6 = “No exertion at all” to 20 = “Maximal exertion”)^45^ at BASELINE and POST. Furthermore, participants completed more detailed questionnaire including the Motion Sickness Assessment Questionnaire (MSAQ), Positive and Negative Affect Schedule (PANAS), and Epworth Sleepiness Scale (ESS) before (BASELINE) and after (POST) exercises. MSAQ was used to determine various dimensions (e.g., gastrointestinal) of motion sickness on a scale from 1 to 9^46^. PANAS was used to assess the effect of centrifugation upon mood on a Likert scale from 1 “not at all” to 5 “very much”^47^. Induced drowsiness was assessed with the ESS (rating from 0 (non-) to 3 “high chance of dozing” in 8 contexts)^48^.

### Statistical analysis

The primary goal of this pilot trial was to determine feasibility and safety of rowing exercises in artificial gravity in a terrestrial environment. The key exploratory endpoint was completion of exercise sessions without medical events such as pre-syncope, emesis, or pain defined as ratings of 4 or more on the Wong-Baker scale. We prospectively defined feasibility as ≤2 of premature training termination due to these events. Given the exploratory nature of the study, we did not perform a formal sample size calculation. However, considering insights from prior research, a sample size of *n* = 12 was deemed appropriate to identify relevant increases in pre-syncope, emesis, or pain. We performed paired *t* tests for cardiorespiratory data, mean questionnaire results (MS, RPE, MSAQ, PANAS, ESS) before and after rowing as well as for mean rower performance values (stroke rate, Watt, power, total distance, total duration) in the centrifuge and control condition.

We applied linear mixed models to determine if there was an effect of condition (CONTROL, SAHC), rotational direction (clockwise: SAHC-CW, counter clockwise: SAHC-CCW), time (Watt-stages), laterality (RIGHT, LEFT), and leg muscle activity (rectus femoris, gastrocnemius, tibialis, vastus lateralis) until stage 8. We used stage 8 (135 W) as the cut-off to ensure sufficient data points per group. Furthermore, we used linear mixed models to compared the effects of condition and time (BASELINE, PRE-EXERCISE, Watt-Stages, POST) upon cardiovascular response (heart rate, blood pressure) and blood lactate results. We determine if there was an effect of condition, laterality (RIGHT, LEFT), and movement phase (CONCENTRIC, ECCENTRIC) upon kinematic data. All statistical tests were conducted using R (version 4.1.2) with *p* < 0.05 assumed as being statistically significant.

## Data Availability

The datasets generated from the study are available from the authors.
